# In this issue of Current Oncology

**Published:** 2007-06

**Authors:** M. McLean

The Editorial Board is pleased to announce that *Current Oncology* is now included in the U.S. National Library of Medicine’s PubMed search engine. PubMed provides access to bibliographic information that spans medline and oldmedline, plus out-of-scope citations (for example, articles on plate tectonics and astrophysics) from certain medline journals, primarily those on general science and chemistry, whose life sciences articles are indexed for medline. Until now, *Current Oncology* has principally been indexed in Excerpta Medica, and so we are pleased to record this development and the related vote of confidence that it reflects.

“Palliative care aims to improve the life of patients and families through the early identification and impeccable management of suffering associated with advanced illness and emphasis on the positive aspects of life ...” So begins the overview from Dr. Neil MacDonald in his article discussing current and past cancer symptom trials and, in particular, their scope and limitations. His hard-hitting conclusions are worthy of study.

Dr. Richard Mould continues his review of significant past events that helped shape modern radiotherapy practices in his review of the management of benign diseases (a favourite examination topic in years gone by).

Drs. Goss, Richardson, and Johnston introduce our new section titled “Canadian Centre Activities” with a review of the National Cancer Institute of Canada Clinical Trials Group map.3 trial, an international breast cancer prevention trial.

And in the Practice Guidelines section, panel reviews of the management of glioblastoma multiforme and the role of alemtuzumab in chronic lymphocytic leukemia are respectively chaired by Dr. Warren Mason and Dr. Christopher Smith.

